# A Combined Clinical and Serum Biomarker-Based Approach May Allow Early Differentiation Between Patients With Minor Stroke and Transient Ischemic Attack as Well as Mid-term Prognostication

**DOI:** 10.3389/fneur.2021.724490

**Published:** 2021-11-08

**Authors:** Johann Otto Pelz, Katharina Kubitz, Manja Kamprad-Lachmann, Kristian Harms, Martin Federbusch, Carsten Hobohm, Dominik Michalski

**Affiliations:** ^1^Department of Neurology, University Hospital Leipzig, Leipzig, Germany; ^2^Institute of Clinical Immunology and Transfusion Medicine, University of Leipzig, Leipzig, Germany; ^3^Institute of Laboratory Medicine, Clinical Chemistry and Molecular Diagnostics, University Hospital Leipzig, Leipzig, Germany; ^4^Department of Neurology, Carl-Von-Basedow-Klinikum Saalekreis, Merseburg, Germany

**Keywords:** biomarker panel, minor ischemic stroke, transient ischemic attack, prognostication, functional impairment

## Abstract

**Background:** Early differentiation between transient ischemic attack (TIA) and minor ischemic stroke (MIS) impacts on the patient's individual diagnostic work-up and treatment. Furthermore, estimations regarding persisting impairments after MIS are essential to guide rehabilitation programs. This study evaluated a combined clinical- and serum biomarker-based approach for the differentiation between TIA and MIS as well as the mid-term prognostication of the functional outcome, which is applicable within the first 24 h after symptom onset.

**Methods:** Prospectively collected data were used for a retrospective analysis including the neurological deficit at admission (National Institutes of Health Stroke Scale, NIHSS) and the following serum biomarkers covering different pathophysiological aspects of stroke: Coagulation (fibrinogen, antithrombin), inflammation (C reactive protein), neuronal damage in the cellular [neuron specific enolase], and the extracellular compartment [matrix metalloproteinase-9, hyaluronic acid]. Further, cerebral magnetic resonance imaging was performed at baseline and day 7, while functional outcome was evaluated with the modified Rankin Scale (mRS) after 3, 6, and 12 months.

**Results:** Based on data from 96 patients (age 64 ± 14 years), 23 TIA patients (NIHSS 0.6 ± 1.1) were compared with 73 MIS patients (NIHSS 2.4 ± 2.0). In a binary logistic regression analysis, the combination of NIHSS and serum biomarkers differentiated MIS from TIA with a sensitivity of 91.8% and a specificity of 60.9% [area under the curve (AUC) 0.84]. In patients with NIHSS 0 at admission, this panel resulted in a still acceptable sensitivity of 81.3% (specificity 71.4%, AUC 0.69) for the differentiation between MIS (*n* = 16) and TIA (*n* = 14). By adding age, remarkable sensitivities of 98.4, 100, and 98.2% for the prediction of an excellent outcome (mRS 0 or 1) were achieved with respect to time points investigated within the 1-year follow-up. However, the specificity was moderate and decreased over time (83.3, 70, 58.3%; AUC 0.96, 0.92, 0.91).

**Conclusion:** This pilot study provides evidence that the NIHSS combined with selected serum biomarkers covering pathophysiological aspects of stroke may represent a useful tool to differentiate between MIS and TIA within 24 h after symptom onset. Further, this approach may accurately predict the mid-term outcome in minor stroke patients, which might help to allocate rehabilitative resources.

## Introduction

Time-sensitive diagnosis of ischemic stroke is essential for patients not only to allow decision making regarding acute treatment, but also to guide the individual diagnostic workup (1). Especially in patients presenting with minor or short-lasting neurological deficits, the differentiation between minor ischemic stroke (MIS) (2) and transient ischemic attack (TIA) is rather challenging. Furthermore, knowledge on the individual diagnoses is essential to initiate rehabilitative programs early after the event with the intention to reduce stroke-related sequelae as best as possible.

According to the widely applied definitions, cerebral magnetic resonance imaging (MRI) is mandatory in these patients to detect neuronal damage in terms of an ischemic lesion (3). However, computed tomography is routinely used as first cerebral imaging method in many countries, since it offers all necessary information for acute treatment decisions. Although a more accurate diagnosis may arise from MRI especially in early phases, access to this technique is typically limited due to costs and availability.

In addition to cerebral imaging, serum biomarkers have been discussed as an option to differentiate between ischemic stroke and TIA (4). So far, many serum biomarkers were examined in the (hyper)acute phase of ischemic stroke with the intention to guide acute treatment decisions like systemic thrombolysis by reliably differentiating ischemic from hemorrhagic stroke (5, 6). However, serum biomarkers alone or in combination have often demonstrated an only moderate to good sensitivity for the differentiation between ischemia and hemorrhage (7). In a more general perspective, the etiology of ischemic stroke is known to be rather complex, ranging for example from cardio-embolic sources, to carotid artery or small vessel disease (8), to rare causes like spontaneous cerebral artery dissection (9), or tumor-associated hypercoagulability (10). Thus, it seems plausible that a single serum biomarker or even a panel that focus on one mechanism cannot cover the variety of aspects involved in stroke pathophysiology.

Furthermore, most biomarker approaches addressed only single scenarios like the differentiation between ischemic and hemorrhagic stroke [e.g., (5)], the prediction of stroke associated complications (11), or the prognostication of functional outcome after ischemic stroke (12). A biomarker-based approach that covers multiple of these scenarios would be easy to use and resource-effective, which would facilitate its translation and acceptance into daily clinical practice.

Considering different pathophysiological aspects of stroke, this study aimed to evaluate a combined clinical- and serum biomarker-based approach within the first 24 h after symptom onset for the differentiation between TIA and MIS and for the prognostication of the functional outcome in MIS patients in a follow-up period of 12 months.

## Methods

### Study Design and Content

**Figure 1:**
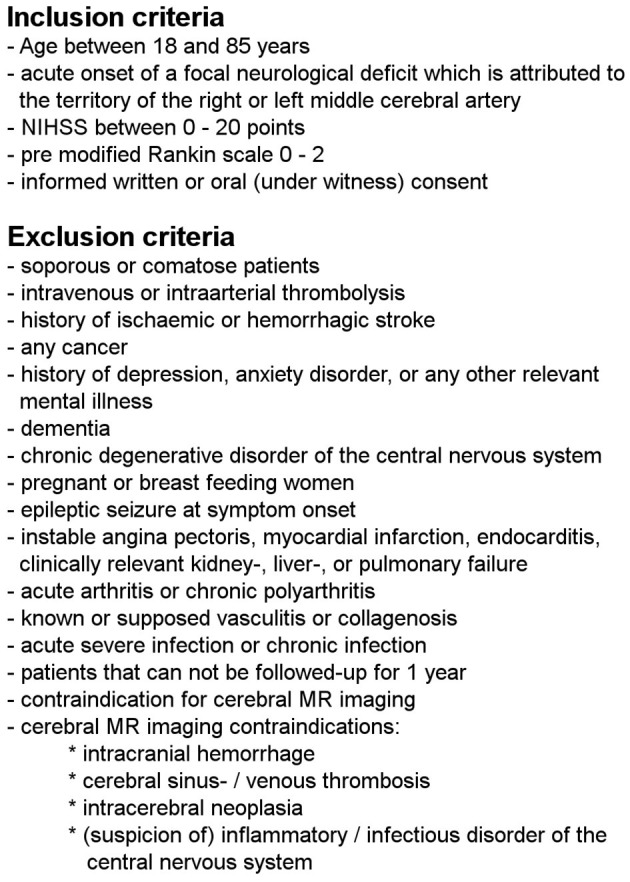
In- and exclusion criteria of the study population in detail.

This work used data from a prospective observational study, which complies with the guidelines for human studies and had been approved by the local ethic committee of the University of Leipzig (approval number 198-08). In- and exclusion criteria are shown in detail in Figure 1. All participants have given informed consent, either in a written or witnessed oral form. Patients were enrolled between 11/2008 and 09/2010 at the Stroke Unit of the Department of Neurology at the University Hospital Leipzig within the first 24 h after symptom onset. Information about symptom onset or last seen well were given by the patients themselves. Patients with evidence for hemorrhage on initial cerebral imaging were excluded.

The pre-hospital functional status and the functional status at admission as well as at months 3, 6, and 12 were assessed by the modified Rankin Scale (pre-mRS, mRS at admission, etc.). The National Institute of Stroke Scale (NIHSS) was used to assess the severity of neurological symptoms at admission. The assessors of the mRS during the follow-up period were unaware of the results of the clinical and para-clinical (imaging, laboratory) baseline examinations.

Blood samples were collected at study enrollment by venipuncture (EDTA, serum and citrate blood, S-Monovette®, Sarstedt AG&Co, Germany) and the following laboratory parameters were measured: leukocyte and platelet count (automated hematology analyzer XE-2100, Sysmex Europe, Germany), C reactive protein (CRP) (latex-enhanced immunoturbidimetric test, cobas® analyzer, Roche Diagnostics, Germany), interleukin 6 (IL-6), neuron specific enolase (NSE) (electrochemiluminescence immunoassay, cobas®), procalcitonine (PCT) (Immunofluorescent assay, Kryptor Immunoanalyzer, Brahms AG, Germany), D-Dimer (latex-enhanced immunoturbidimetric test, BCS® coagulation analyzer, Siemens, Germany), activated partial thromboplastin time (aPTT), fibrinogen, prothrombin time (clotting-based; PT, given as activity percentage based on the Quick method involving a standard curve based on dilutions of normal plasma, BCS®), and antithrombin (chromogenic, BCS®).

Only CE-IVD-certified laboratory tests approved for diagnostic use were applied, and all analytical procedures were performed according to the manufacturer's instruction. Analysis was performed in the Institute of Laboratory Medicine, Clinical Chemistry and Molecular Diagnostics, University Hospital Leipzig.

The serum levels of matrix metalloproteinase-9 (MMP-9), tissue inhibitor of matrix metalloproteinase 1 (TIMP-1), and hyaluronic acid were measured by certified enzyme-linked immunosorbent assays (designed by Cloud-Clone Corp., Houston, United States of America; assembled by Uscn Life Science Inc., Wuhan, People's Republic of China).

All study participants underwent cerebral 1.5 Tesla MRI upon enrollment in the study and at day 7 ± 1. Based on cerebral MRI and duration of symptoms, patients were classified to either an ischemic stroke (evidence of an acute ischemic lesion in the diffusion weighted imaging sequence in at least one MRI *and/or* a symptom duration of more than 24 h) or a TIA (no evidence of an acute lesion in the diffusion weighted imaging sequence of both MRI *and* a symptom duration of <24 h), according to Sacco et al. (3).

### Statistical Analysis

For statistical calculations the IBM SPSS Statistics Package Version 25 (IBM Corp., Armonk, NY, USA) was used. After descriptive analyses, statistical significance between groups was assessed by chi square test for categorical variables and by Mann-Whitney U test for interval-scaled parameters. Bonferroni-Holm correction was applied to consider multiple testing. Based on pathophysiological considerations (7) we calculated forward logistic regression analyses with MIS vs. TIA and excellent (mRS 0–1) vs. non-excellent (mRS ≥ 2) functional outcome after 3, 6, and 12 months as dependent variables and different combinations of clinical data (NIHSS, age) and laboratory parameters (with at least one parameter from each of the four domains) as predictor variables to obtain the sensitivity, specificity, and area under the curve (AUC) of the applied models.

## Results

**Figure 2:**
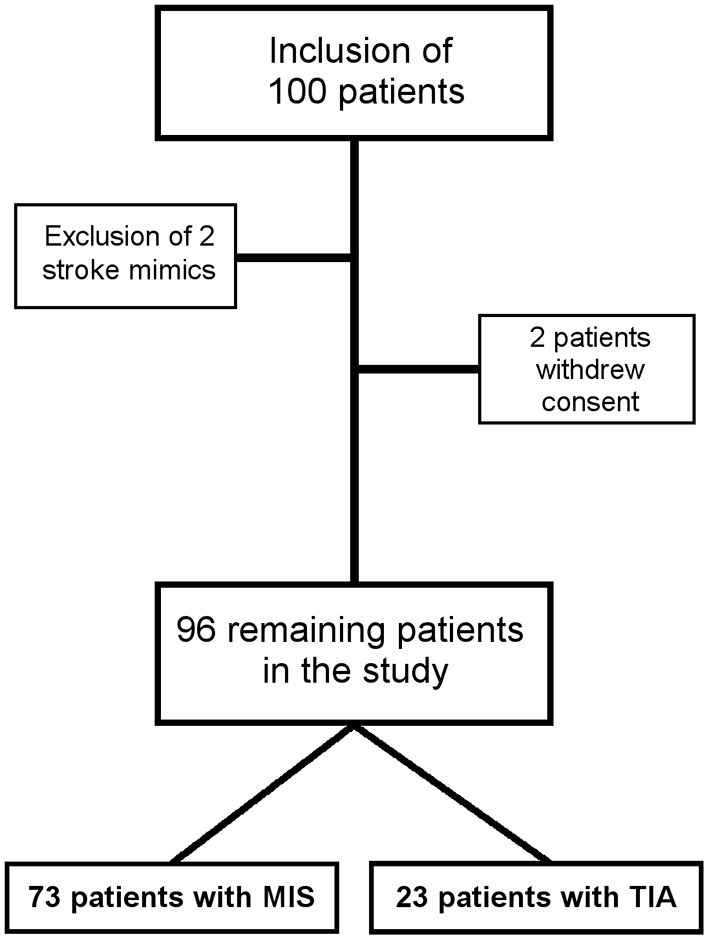
Flow chart for the study. Minor ischemic stroke MIS, transient ischemic attack TIA.

Data from 100 patients were used for the study. Two patients with stroke mimics (one patient with a peripheral facial nerve paresis, one patient with meningitis) and two patients who withdraw their consent were excluded (Figure 2). Thus, data from 96 patients (mean age 64 ± 14 years) were included in the final analysis, while 23 TIA patients (NIHSS at admission 0.6 ± 1.1) were compared with 73 MIS patients (NIHSS at admission 2.4 ± 2.0). Detailed baseline demographic and clinical data of patients are shown in Table 1 and, with the exception of a higher NIHSS and a higher mRS at admission for patients with MIS, were almost similar between patients with MIS and patients with TIA. Etiology of MIS or TIA is shown in Table 2; while carotid artery disease with at most moderate stenosis (<70% NASCET) was more prevalent in patients with MIS, patients with TIA were diagnosed more often as of cryptogenic etiology. Laboratory panel parameters are given in Table 3 as mean values and as the number and percentage of values outside the local laboratory reference intervals. Briefly, we found no significant differences between both groups including all laboratory parameters.

**Table 1:** Baseline demographic and clinical data of patients with transient ischemic attack and minor ischemic stroke.

	**Patients with MIS**	**Patients with TIA**	***p***
	**(*n* = 73)**	**(*n* = 23)**	
Age in years	64.6 ± 12.8	63.4 ± 16.3	0.919
Female/Male	34/39	11/12	
NIHSS at admission	2.4 ± 2.0	0.6 ± 1.1	<0.001^#^
Pre-mRS	0.1 ± 0.3	0.3 ± 0.7	0.404
mRS at admission	1.6 ± 1.3	0.6 ± 0.8	0.002^#^
mRS at 3 months	0.9 ± 1.1	–	–
mRS at 6 months	0.9 ± 1.0	–	–
mRS at 12 months	0.9 ± 1.2	–	–
Arterial hypertension	55 (75.3 %)	16 (69.6 %)	0.582^*^
Diabetes mellitus	11 (15.1 %)	4 (17.4 %)	0.789^*^
Current smoking	16 (22.0 %)	3 (13.0 %)	0.352^*^
Hyperlipidemia	22 (30.1 %)	8 (34.8 %)	0.675^*^

*MIS, minor ischemic stroke; TIA, transient ischemic attack; mRS, modified Rankin Scale*.

# *Mann-Whitney U test*.

* *Chi square test*.

**Table 2:** Etiologies of patients with minor ischemic stroke and transient ischemic attack.

**Etiology**	**Patients with MIS**	**Patients with TIA**	***p***
	**(*n* = 73)**	**(*n* = 23)**	
Carotid artery disease with at most moderate stenosis (<70% NASCET)	41 (56.2%)	1 (4.3%)	<0.001
Carotid artery disease with high grade stenosis (≥70%)	7 (9.6%)	4 (17.4%)	0.301
Cardio-embolic	15 (20.5%)	1 (4.3%)	0.069
Small vessel disease	3 (4.1%)	1 (4.3%)	0.960
Spontaneous cervical artery dissection	3 (4.1%)	0	–
Cryptogenic	4 (5.5%)	16 (69.6%)	<0.001

*Groups were compared using chi square test. MIS, minor ischemic stroke; TIA, transient ischemic attack*.

**Table 3:** Comparison of biomarkers between patients with minor ischemic stroke and transient ischemic attack.

	**Patients with MIS**	**Patients with TIA**	***p***
	**(*n* = 73)**	**(*n* = 23)**	
**Coagulation System**			
Fibrinogen (g/L)	3.3 ± 0.9	2.8 ± 0.7	0.018
	20 (27.4%)	1 (4.3%)	0.020^#^
D-Dimer (mg/L)	0.98	0.70	0.644
	25^th^ percentile: 0.33	25^th^ percentile: 0.22	
	75^th^ percentile: 0.81	75^th^ percentile: 1.08	
	37 (50.7%)	10 (43.5%)	0.547^#^
Antithrombin (%)	92.7 ± 11.6	95.6 ± 10.2	0.259
	3 (4.1%)	0 (0%)	–
Thrombocyte count (10^9^/L)	232 ± 63	214 ± 60	0.313
	15 (20.5%)	3 (13%)	0.421^#^
aPTT (s)	31.3 ± 4.2	30.0 ± 3.8	0.166
	7 (9.6%)	1 (4.3%)	0.428^#^
Prothrombin time (%)	99 ± 19	104 ± 10	0.317
**Inflammation**			
Leucocyte count (10^9^/L)	7.9 ± 2.5	8.0 ± 2.0	0.356
	20 (27.4%)	6 (26.1%)	0.902^#^
Interleukin 6 (pg/ml)	10.2 ± 10.2	6.9 ± 6.7	0.051
	38 (52.1%)	7 (30.4%)	0.070^#^
CRP (mg/L)	5.4	3.0	0.237
	25^th^ percentile: 1.2	25^th^ percentile: 1.0	
	75^th^ percentile: 5.2	75^th^ percentile: 3.7	
	19 (26.0%)	4 (17.4%)	0.397^#^
PCT (ng/ml)	0.06 ± 0.03	0.09 ± 0.09	0.930
	46 (63.0%)	18 (78.3%)	0.176^#^
**Neuronal Damage**			
NSE (ng/ml)	26.7 ± 13.0	21.9 ± 6.7	0.038
	64 (87.7%)	17 (73.9%)	0.113^#^
**Markers of the Extracellular Compartment**			
MMP-9	232 ± 158	251 ± 179	0.776
TIMP-1	245 ± 159	212 ± 171	0.189
Hyaluronic acid	96.1 ± 56.6	110.6 ± 76.1	0.533

*Non-parametric testing (Mann-Whitney U test) was applied for intergroup comparison with correction for multiple testing (Bonferroni-Holm correction), resulting in a corrected significance level of p = 0.0036. Number and percentage of values outside the local laboratory reference intervals are given in the second row for the respective parameter with statistical significance being tested between groups with chi square test (indicated by #). MIS, minor ischemic stroke; TIA, transient ischemic attack; aPTT, activated partial thromboplastin time; CRP, C reactive protein; PCT, procalcitonin; NSE, neuron specific enolase; MMP-9, matrix metalloproteinase-9; TIMP-1, tissue inhibitor of matrix metalloproteinase 1*.

**Figure 3:**
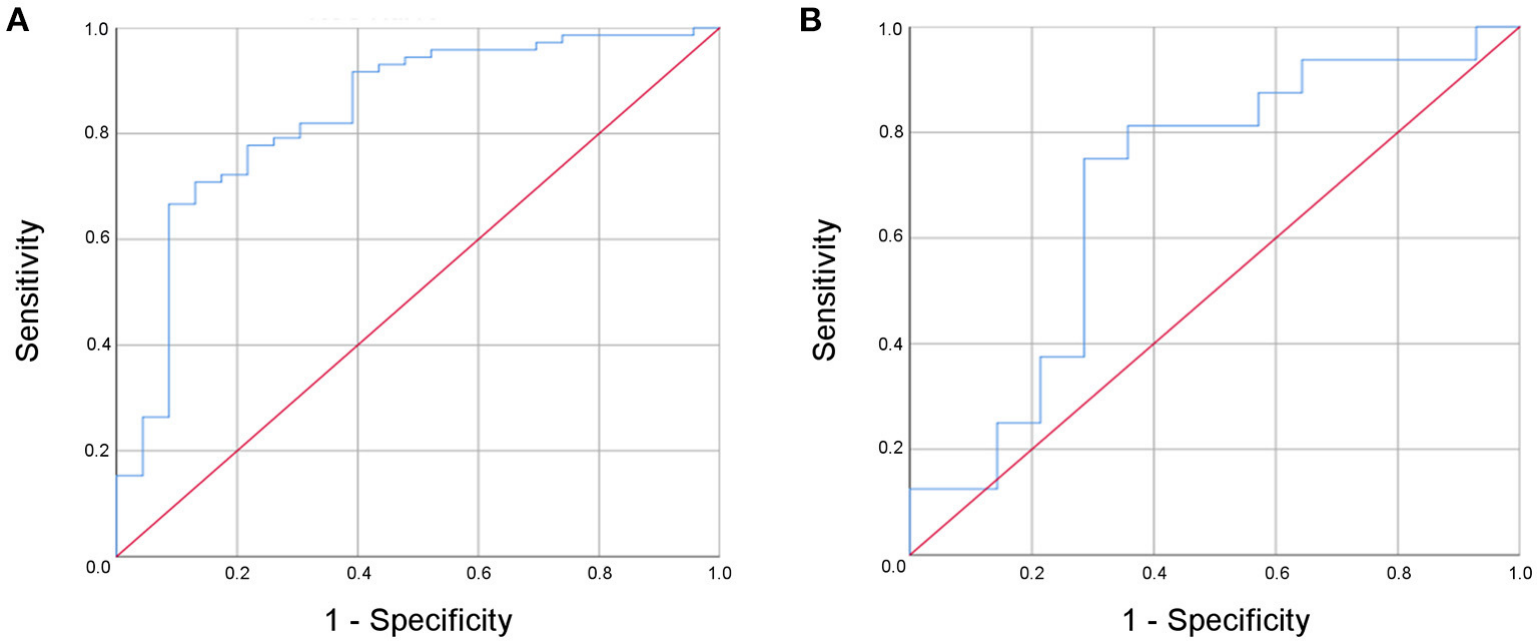
Receiver operated curve (ROC) analysis of the multi-modal biomarker panel for the differentiation between minor ischemic stroke and transient ischemic attack for all patients **(A)** and for patients with complete recovery upon admission to the stroke unit **(B)**.

In a binary logistic regression analysis with ischemic stroke or TIA as the dependent variable the combination of NIHSS at admission, fibrinogen, antithrombin, CRP, MMP-9, hyaluronic acid, and NSE was found to be best associated with MIS, resulting in a sensitivity of 91.8% and a specificity of 60.9% (AUC 0.84, 95% confidence interval 0.74–0.94) (Figure 3A). An increased NIHSS at admission doubled the risk for the patient to have suffered an ischemic stroke (odd ratio 2.0; confidence interval 1.3–3.3; Table 4). Selecting any biomarker of inflammation while excluding the others did not change sensitivity of this model relevantly: CRP 91.7%, IL-6 88.7%, PCT 93.1%, and leucocyte count 93.0%, while specificity was also comparable: CRP 60.9%, IL-6 52.2%, PCT 60.9%, and leucocyte count 56.5%. Focusing only on patients with complete recovery of neurological symptoms (NIHSS 0) upon admission to the stroke unit, this panel resulted in a still acceptable sensitivity of 81.3% and a specificity of 71.4% (AUC 0.69, 95% confidence interval 0.49–0.89) for the differentiation between MIS (*n* = 16) and TIA (*n* = 14) (Figure 3B).

**Table 4:** Odds ratios with confidence intervals for the NIHSS score and each laboratory parameter that was included into the model to differentiate between patients with minor ischemic stroke and transient ischemic attack within 24 h after symptom onset.

	**Odds ratio**	**Confidence interval**
NIHSS	2.04	1.27–3.29
Fibrinogen	2.28	0.77–6.76
Antithrombin	0.98	0.93–1.04
CRP	0.97	0.84–1.13
NSE	1.06	0.98–1.16
MMP-9	1.0	1.0–1.0
Hyaluronic acid	1.0	0.99–1.01

*CRP, C reactive protein; NSE, neuron specific enolase; MMP-9, matrix metalloproteinase-9; NIHSS, National Institute of Health Stroke Scale*.

**Figure 4:**
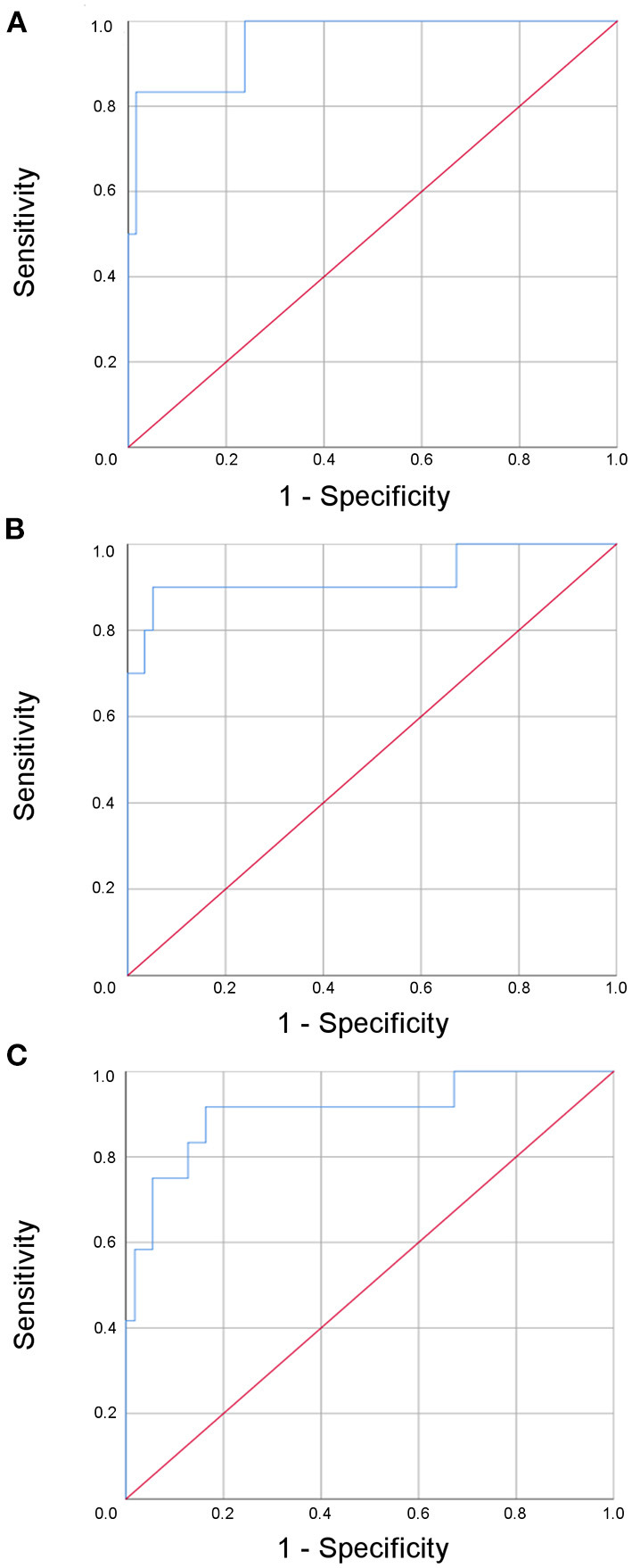
Receiver operated curve (ROC) analysis of the multi-modal biomarker panel for the prediction of an excellent outcome after three **(A)**, six **(B)**, and twelve **(C)** months for patients with minor ischemic stroke.

By adding age, this multi-dimensional approach yielded remarkable sensitivities of 98.4, 100, and 98.2% with respect to the time points investigated within the first 12 months after the event for predicting an excellent outcome (mRS 0 or 1) (Figure 4). However, the specificity was moderate and decreased over time (83.3, 70, and 58.3%; AUC 0.96 (95% confidence interval 0.88–1.0), 0.92 (95% confidence interval 0.80–1.0), 0.91 (95% confidence interval 0.80–1.0).

## Discussion

This pilot study provides robust evidence that a combined clinical- and serum biomarker-based approach, which covers different pathophysiological aspects of stroke and is obtained early after the event, might help to differentiate between MIS and TIA. This is of interest, as early differentiation appears difficult in clinical practice and usually depends on an additional cerebral MRI. Moreover, the same approach was shown to accurately predict an excellent mid-term outcome in patients suffering from MIS.

Although many serum biomarkers and their combinations have been evaluated in the setting of an acute ischemic stroke, a “troponin of the brain,” i.e., a highly sensitive and specific serum biomarker indicating an acute ischemic damage, is still lacking. Hence, the diagnosis of an ischemic stroke is currently linked to the detection of a persistent and not only transient neuronal damage either by cerebral imaging in terms of an ischemic lesion or by persisting neurological symptoms for more than 24 h (3). Besides the differentiation between ischemic and hemorrhagic stroke within the (hyper)acute phase of stroke (5, 6), serum biomarkers might also be helpful in predicting complications of stroke like pneumonia (11, 13), in functional prognostication (12, 14, 15), as well as in the allocation of diagnostic and rehabilitative resources. However, so far, no single biomarker or panel of biomarkers succeeded translation from bench to bedside, i.e., to facilitate diagnosis, treatment, or prognostication (7). Furthermore, highly elaborated multistep approaches in large samples have failed (16–18). One reason for this failure might be the variety of stroke etiologies and the complex pathophysiological mechanisms occurring during early stages after the event (8, 19).

Consequently, recent reviews discussed the potential use of biomarker panels that would cover several relevant pathophysiological aspects of stroke. In their review, Baez et al. proposed different combinations of biomarkers comprising cellular (glial or neuronal) components, extracellular components, and the coagulation system (20). Here, we combined clinical data with serum biomarkers involving the coagulation system, inflammation, neuronal damage, and the extracellular compartment. In our study the combination of the NIHSS, fibrinogen, antithrombin, CRP, MMP-9, hyaluronic acid, and NSE within 24 h after symptom onset was found to be associated with MIS, resulting in an accuracy of 0.84. Further, in the subgroup of patients presenting with a complete recovery of neurological symptoms at admission to the stroke unit, this combined approach still resulted in an accuracy of 0.69.

Remarkably, by adding age, the same multi-modal approach accurately predicted an excellent mid-term outcome in patients with MIS, too. Therefore, this proposed approach might facilitate further decisions in the diagnostic work-up (e.g., the need and timing of MRI, or the length of monitoring on a stroke unit) and in allocation to rehabilitation.

So far, biomarkers were optimized with respect to different scenarios (7, 14). Thus, the composition of biomarker panels does not only greatly vary within the same scenario (e.g., the differentiation between ischemic and hemorrhagic stroke) but even more between different scenarios. Hence, the here applied clinical- and biomarker-based approach would be a pragmatic compromise for two relevant scenarios. In particular, this multi-modal biomarker approach might be cost-effective in settings where cerebral MRI is not available in time or causes of great costs. Of course, acute treatment decisions like systemic thrombolysis will still be based on cerebral imaging as for instance CT, which is widely available and sufficient to rule out an intracranial bleeding (1). However, in clinical routine, the current diagnostic work-up of patients suffering from a focal neurological deficit with sudden onset do not depend on a confirmed ischemic stroke by cerebral MRI or a symptom duration of more than 24 h. Due to the high sensitivity of the here described combined clinical and serum biomarker-based approach, a future paradigm might allow the diagnosis of a biomarker-positive ischemic stroke while the confirmatory cerebral MRI could be omitted. Furthermore, the same biomarker panel might support decisions concerning the allocation of patients with MIS to rehabilitation at all, as well as to the timing and to the kind of rehabilitation (early vs. delayed, in- vs. out-patient rehabilitation).

This study has some limitations. First, although data collection was performed in a prospective manner, this study followed a retrospective data analyses, which limits generalization of the findings. Second, due to the relatively small sample size, the combined clinical- and serum biomarker-based approach needs to be confirmed in a larger cohort of patients with MIS and TIA, preferably with the use of validation groups. However, statistical analyses without pre-specified parameters carry the inherent risk for an over-optimization of the statistical model, and, therefore, an overestimating effect size. Noteworthy, validation studies can yield less accurate results than the initial study (16, 18). Third, blood samples were collected at a single time point within the first 24 h after symptom onset. Release kinetics of biomarkers may naturally differ, especially within the acute phase of stroke. For example, levels of NSE were found to demonstrate a biphasic course with a first rise within 3 h followed by a decrease and secondary increase until day 5 (21). Varying levels within the first 25 h were also described for MMP-9 and TIMP-1 in experimental stroke (22). Thus, a smaller time window for the collection of blood samples, blood sampling at specified time points, or repeated collections might increase accuracy. Further, future studies may also include novel biomarkers as for instance neurofilament light chain, which has shown an association with the long-term outcome after stroke (23), while its relevance in the acute and short-term course is still pending (24). Fourth, in MIS patients the etiology of the event was very heterogeneous and the relatively small sample size excluded further subgroup analyses. As a rule of thumb, there should be at least 20 patients for every predictor variable in a binary logistic regression analysis which would have resulted in the inclusion of at least 140 patients in this study. Thus, this study might have been underpowered.

On the other side, one strength of this study is the in-depth characterization of patients with TIA based on two negative MRI examination at the time point of study inclusion and day 7. Moreover, we addressed two relevant scenarios with the same clinical- and biomarker-based approach.

## Conclusion

This pilot study provides evidence that the NIHSS together with a multi-modal panel of serum biomarkers covering pathophysiological aspects of stroke represents a promising tool to differentiate between patients with MIS and TIA within the first 24 h after symptom onset. Furthermore, only by adding age, the same approach accurately predicted an excellent mid-term outcome in patients with MIS. Assuming that these findings can be confirmed in larger cohorts of stroke patients, the emerging paradigm of a biomarker-positive ischemic stroke might allow a more focused diagnostic workup and early planning of rehabilitative programs.

## Data Availability Statement

The raw data supporting the conclusions of this article will be made available by the authors upon reasonable request.

## Ethics Statement

The study complies with the guidelines for human studies and had been approved by the Local Ethics Committee of the University of Leipzig (approval number 198-08). All participants have given informed consent, either in a written or witnessed oral form.

## Author Contributions

DM and CH conceived the underlying investigation, while JP conducted the part resulting in the present study. DM, CH, MF, JP, and KK collected data. KH and MK-L contributed to data analysis. JP and DM performed data analyses and wrote the paper. All authors provided critical revisions to the manuscript.

## Conflict of Interest

The authors declare that the research was conducted in the absence of any commercial or financial relationships that could be construed as a potential conflict of interest.

## Publisher's Note

All claims expressed in this article are solely those of the authors and do not necessarily represent those of their affiliated organizations, or those of the publisher, the editors and the reviewers. Any product that may be evaluated in this article, or claim that may be made by its manufacturer, is not guaranteed or endorsed by the publisher.
